# Genome editing technologies: CRISPR, LEAPER, RESTORE, ARCUT, SATI, and RESCUE

**DOI:** 10.17179/excli2020-3070

**Published:** 2021-01-04

**Authors:** Senay Görücü Yilmaz

**Affiliations:** 1 Department of Nutrition and Dietetics, Gaziantep University, Gaziantep, Turkey 27310

**Keywords:** genome editing, bioengineering, genome editing technologies, gene therapy

## Abstract

Genome editing technologies include techniques used for desired genetic modifications and allow the insertion, modification or deletion of specific DNA fragments. Recent advances in genome biology offer unprecedented promise for interdisciplinary collaboration and applications in gene editing. New genome editing technologies enable specific and efficient genome modifications. The sources that inspire these modifications and already exist in the genome are DNA degradation enzymes and DNA repair pathways. Six of these recent technologies are the clustered regularly interspaced short palindromic repeats (CRISPR), leveraging endogenous ADAR for programmable editing of RNA (LEAPER), recruiting endogenous ADAR to specific transcripts for oligonucleotide-mediated RNA editing (RESTORE), chemistry-based artificial restriction DNA cutter (ARCUT), single homology arm donor mediated intron-targeting integration (SATI), RNA editing for specific C-to-U exchange (RESCUE). These technologies are widely used from various biomedical researches to clinics, agriculture, and allow you to rearrange genomic sequences, create cell lines and animal models to solve human diseases. This review emphasizes the characteristics, superiority, limitations, also whether each technology can be used in different biological systems and the potential application of these systems in the treatment of several human diseases.

## Introduction

Genome editing is a genetic engineering initiative by inserting, deleting, modifying, or replacing DNA into the genome of a living organism. In previous technologies, the genetic material was randomly inserted into the host genome. Since this randomness has the potential to disrupt or alter other genes in the organism, new solutions have been sought. Nowadays, the insertion process can be carried out specifically to the target. Through specifically targeted techniques, off-target effects are reduced and also allow for the regulation of certain sequences within a known genome. In an organism, it has been made possible to cure a specific genetic disease by adding a functional gene to replace the defective gene. To understand the advances in gene-editing techniques, it is necessary briefly to comment on the techniques before CRISPR. Recognizers and degrading enzymes must be found to ensure target specificity. Restriction enzymes used in the process of specific target recognition are called "molecular scissors." These molecular scissors cleave the DNA into specific recognition sequences known as cleavage sites or nearby regions. The use of endonucleases and exonucleases is common for cleavage regulation. These nucleases have two subcategories as deoxyribonucleases and ribonucleases. Specifically produced nucleases can be programmed according to the purpose. Nucleases form double-strand breaks (DSBs) in the genome of interest and are then repaired by cellular mechanisms. These repair mechanisms that give the techniques their name are failure-prone nonhomologous end-joining (NHEJ) and error-free homology directed repair (HDR). Thus, they can cause the addition, deletion or alteration of nucleotide/nucleotides in the target sequence (Helena et al., 2018[47]). Mutations caused by repair mechanisms can disrupt, remove or repair the defective region in genes. So what is our main purpose in using these techniques? The process that starts with the treatment of diseases in genome technologies continues until the breeding and productivity of plants (Friedrichs et al., 2019[34]) and animals (Zhao et al., 2019[142]). Researchers are developing new tools involving gene/genome editing to prevent and treat diseases in humans. Genome editing tools have the potential to treat diseases such as cystic fibrosis (Hodges and Conlon, 2019[50]), DMD (Fernandez-Ruiz, 2020[32]), and diabetes (Balboa et al., 2019[13]). The main goal in genome editing is to make it possible to treat genetic-based diseases. Therefore, choosing the appropriate treatment depends on genome disorder to be treated. The best way to correct the genome depends on the target and/or the type of damage and the size of the target. The unknown target and what kind of correction will be encountered after genetic modifications may be disappointing at the end of the process. Target molecules in the application of these techniques can be DNA, RNA, mitochondrial genome or protein. In addition to these, the target tissue or biological resources can be germline, somatic cell, stem cells or embryos from different lineages. Different techniques can be used alone or in combination, and understanding the potential of these techniques is important for the effectiveness of genome editing technologies. The programming of the genome has greatly accelerated the gene-editing process in many areas and uniquely enables researchers to perform the maneuver they want on the genome. Preclinical studies on genome editing focus on the correction of single-gene diseases, immunotherapies, cancer, viral infections and cardiovascular diseases. While some of these techniques are applied directly to the patient, some of them are still in the clinical trial phase. Here, we review six genome editing technologies and discuss applications that can be done using a variety of organisms and gene editing tools.

## Mechanisms of Genome Editing Tools

### CRISPR-CAS SYSTEM: naturally occurring genome editing system

CRISPR is a breakthrough that has caught the attention of almost every scientist and imagined what could be done. While this technology looks complex, its basis is clear, and it is effective in correcting genes. In other words, we can say that CRISPR technology is based on searching for a specific piece of DNA in a cell and changing this piece of DNA (Rath et al., 2015[105]) and can turn genes on or off without changing the sequences in its target (Mrowka et al., 2018[93]). CRISPR technology can enable us to treat or prevent many diseases. When we go to a step further, we can reprogram genomes within the framework of ethical rules against congenital or acquired diseases with this technology in the ethical framework. The biggest support for technology is the Cas (Cellular apoptosis susceptibility) enzyme, found in bacteria and a CRISPR partner used in defense against viruses (White et al., 2015[131]). Cas9 can guide access to specific locations in the genome by searching for a short RNA (Hsu et al., 2014[54]) and can be programmed to bind to CRISPR (Wilkinson et al., 2019[134]). Using the CRISPR-Cas system, DNA sequences and proteins in the endogenous genome can be more easily edited or modulated in any selected organism. In addition to being simple and measurable, the genetic repair mediated by Cas-9 also proves the reason for the links between genetic variations and biological phenotypes. The importance of CRISPR, found during the examination of the bacterial genome, comes from its matching with some virus sequences. When bacteria become infected with the virus, they store the DNA fragments of the virus in the CRISPR sequence and thus form a bacterial index (Karginov and Hannon, 2010[59]). In this way, they develop an adaptive immune system. By these mechanisms, bacteria cannot get rid of viral infection with this process alone. Also, the bacterial virus must lyse. At this point, RNAs synthesized from CRISPR sequences (CRISPR RNA-crRNA) and Cas proteins are required. crRNAs provide synthesis using sequences that help identify the virus, and when the virus enters the cell, it performs the recognition process by matching the target sequence with single-stranded RNAs (Hille and Charpentier, 2016[49]). Then the main mission is to destroy the virus, and this task is performed with the CRISPR-Cas system. As an RNA-guided protein, Cas's goal is to break down double-strand DNA and bind to the virus genome with CRISPR in the cell (Jiang and Doudna, 2017[58]). As a result, Cas cuts the binding sites in its target and deactivates the virus (Hryhorowicz et al., 2017[52]). Meanwhile, the bacterium makes copies of the virus genome to add to own account (Gebre et al., 2018[37]). It synthesizes RNA and protein using the sequence it has copied. In this way, it develops an adaptive immune system mechanism that targets and destroys viruses that invade the cell. This is important because this mechanism in bacteria can be used to mediate the human genome. The Cas protein does not bind randomly to target DNAs. It can bind completely to the desired target through crRNAs and mRNAs. According to the technology applied today, the most widely used Cas protein is Cas9. For Cas9 to be functional, the desired sequence must be added and at the same time prevented from truncating the target DNA. After that, the normal protein synthesis mechanism works and proteins are produced from DNA in the presence of polymerase. The polymerase slides along the DNA chain and scans the entire DNA. Thus, protein synthesis takes place. Cas9 is placed in the desired site on the DNA with the crRNA. In this way, the polymerase is prevented from slipping on the DNA chain and the production of the protein for the target gene is prevented. In a sense, the gene is silenced or inactivated. As a result, genes can be activated or vice versa completely silenced with the CRISPR-Cas system. There are many reviews and researches on how the CRISPR-Cas system works and its history. The subject of interest is the contribution of CRISPR technologies to the solution process of human diseases and their applications in other biological systems.

### CRISPR APPLICATIONS: technical approaches with key human diseases 

The first major steps towards finding solutions to human diseases with CRISPR were taken by two research groups. Using this system, the researchers conducted an *in vitro *study on mice. In this model, the cause of DMD was the hotspot mutation in the dystrophin gene and the dystrophin reading frame in the 45-55 exons of the gene was restored using this system (Long et al., 2014[78]; Ousterout et al., 2015[100]). At the end of the study, DMD mutations were corrected by 62 %. Thus, targeting the responsible gene in genetic diseases caused by a single gene and mutation, correcting the mutation in the disease and alleviating the clinical manifestations of the disease seems to be easier compared with multifactorial diseases. The mutations of genes whose genetic basis is changed but, which constitute the center of the disease and are considered to affect the disease, should be corrected by this mechanism and the results should be evaluated. The method is advantageous over other systems. Because it is easy to work, results are obtained in a very short time, and it is made using the organism's mechanisms. The goal today is to correct multiple genes and mutations simultaneously or at different times. 

The CRISPR-Cas system is an important therapeutic target used to understand the mechanism of known genetic diseases, create cell and animal models, and mimic diseases. Genome editing based therapy can provide restoration of gene function or repair of mutation. The most easily applicable genome editing source is SNPs. There are several approaches with different strategies for SNP repair. Considering the number of bases in the DNA and the Watson-Crick match, we can replace the A, T, C, G nucleotides with 12 possibilities. Besides, we can also delete and insert more than one nucleotide as in the deletion of the 4 nucleotides in the HEXA gene that caused Tay-Sachs disease (Min et al., 2019[90]) or exons in DMD (Gadalla et al., 2015[36]; Tremblay et al., 2016[122]). What is the success rate in repairing SNPs when such a process is implemented? It largely depends on choosing the right method. Choosing the right method allows for avoiding the editing of unwanted points in the genome. The precision of this arrangement may only be possible if a single cell is involved. However, additional applications are needed for the specificity of the method. The CRISPR-Cas system has advantages such as high targeted mutation rate, less cost, simplicity, and high multiplex location editing compared to traditional genome editing tools including transcription activator-like effector nucleases (TALENs) and zinc-finger nucleases (ZFNs). This system also has negative effects such as creating unwanted mutations in non-target areas. We can classify these effects in the CRISPR-Cas system as targeted (targeting efficiency) and non-targeted (undesirable). 

One of the additional applications to be made to eliminate these effects in the CRISPR-Cas9 system is the use of single-guide RNA (sgRNA)/ligand-dependent ribozymes called aptazime used in plants (Kundert et al., 2019[71]). Unwanted mutations in non-human organisms can be prevented with this method. It can also be used as an alternative method to significantly reduce the frequency of non-target mutations. When the CRISPR-Cas system is applied, the most common result is insertion/deletions (Indel mutations). For example, in the study where a healthy gene was added to a gene responsible for hypertrophic cardiomyopathy via the CRISPR system, the healthy gene was rejected by human embryos. This result showed the researchers that this repair was not a solution, and they suggested that homology repair is required to treat cardiomyopathy and some other inherited genetic diseases (Ma et al., 2017[80]). Avoiding indel mutations is extremely important in gene therapy applications, as these genomic or chromosomal imbalances can lead to cancer (Rayner et al., 2019[106]; Zhan et al., 2019[139]) and other genomic damage (Ghosh et al., 2019[38]). Can this problem be overcome in CRISPR applications used in cancer therapy? Looking at the live system, we see that CRISPR is used to treat two cancer patients with multiple myeloma and sarcoma (https://www.npr.org/sections/health-shots/2019/04/16/712402435/ First U.S. Patients Treated With CRISPR As Human Gene-Editing Trials Get Underway). Scientists know that there should be a limit to this kind of promising work. However, it is also possible to see news whose ethical dimensions are discussed and the embryo is interfered with (Cyranoski, 2020[28]). It is still difficult to insert target DNA into the CRISPR-Cas system used in the treatment of embryo intervention, cancer, DMD or similar diseases. Today, one of the most effective solutions is the transposons. The CRISPR-associated transposase found in *Cyanobacteria Scytonema hofmanni* (ShCAST) catalyzes RNA guided DNA transposition. Its effectiveness is close to 80 % (Strecker et al., 2019[115]). Another important issue in the CRISPR system is whether the target is single or double-strand DNA. This technique has been used for single-strand DNA. 

This doesn't mean we can't use it for dsDNA. By targeting both dsDNA (DiNapoli et al., 2020[29]) and ssDNA (Bai et al., 2020[12]) templates in cells, experimental animals (Pineault et al., 2019[102]), plants (Mao et al., 2019[87]), model organisms (Bai et al., 2020[11]) and even humans (Ma et al., 2015[79]; Moon et al., 2019[91]), we can regulate insertions, deletions, replication, migration, transformation, copy number loss or gain (Anzalone et al., 2019[6]) with the CRISPR system. Different Cas enzymes are preferred according to the mechanisms by which they are effective in all these biological structures.

### CAS PROTEINS: Essential for comparative genomic and functional characterization of the adaptive immune system

 Gene's editing occurs via mechanisms in which different Cas types are involved. There are about 45 natural Cas protein families divided into eight subtypes, and each family contains 20 proteins. It is divided into three basic classes as I, II, and III (Table 1(Tab. 1)). These classes are also named alphabetically within themselves (for example, I-A, II-B, and III-A) (Zhang et al., 2014[140]). These genes associated with Cas proteins are named as Cas1, 2, 3, and 4 (Haft et al., 2005[43]). There is a typical operon organization for Cas type according to bacteria species. The *Streptococcus thermophilus* has got three Cas genes as Cas9, Cas1, and Cas 2 and *Escherichia coli *K12 has got six Cas genes as Cas3, Cas7, Cas5, Cas6, Cas4, Cas1, Cas2 (Koonin et al., 2017[68]; Makarova and Koonin, 2015[84]). The biggest problem in the literature is the classification of Cas protein types. Cas1 (is a metal-dependent DNA-specific endonuclease and produces dsDNA fragments (Wiedenheft et al., 2009[133]). Cas 1 is part of the Cas-Cas2 complex, and Cas2 is a dsDNase (Nam et al., 2012[96]) and essential for spacer acquisition in the CRISPR system (Nuñez et al., 2014[98]). The importance of Cas1 was understood by increasing sensitivity to DNA damage and impaired chromosomal segregation as a result of gene deletion in *E. coli* (Babu et al., 2011[10]). The classification of Cas1 and Cas 2 genes: Class 1 / Type I, II, III, and U (Makarova and Koonin, 2015[84]). The Cas genes are located in Type I (Cas1, Cas2, Cas3', cas4, Cas5, Cas6, Cas7, Cas8), II (Cas1, Cas2, Cas4, Cas9), III (Cas1, Cas2, Cas5, Cas7, and Cas10), and U (Cas5, Cas7, Cas8) with the other genes (Csn2, RNAse III, SS) (Makarova et al., 2013[86]) (Table 1(Tab. 1)).

These proteins are responsible for the ability of the CRISPR-mediated immune system in bacteria to adapt to new viral infections (He et al., 2018[46]). Type II CRISPR-Cas systems use a double target mechanism that includes RNA-DNA to eliminate invading pathogens. Thus, it checks the strength of CRISPR immunity. The distinction between the self and the dynamics of Cas10, Type III CRISPR-Cas immunity governs the discrimination between self and extra-self in non-self (Wang et al., 2019[127]). To better understand the CRISPR mechanism among these systems, it is necessary to grasp the importance of the Cas 9 protein. CRISPR-Cas is the adaptive antivirus immune system found in many bacteria. The system injects the foreign DNA fragments into the CRISPR cassettes, then copies the CRISPR sequences containing the spacers and processes them to make a guide CRISPR RNA specifically trying to target and cleave the genome of the cognate virus or plasmid. The importance of Cas proteins emerges here. Because these proteins are quite diverse and are required for different stages of processing transcripts of CRISPR loci, such as cleavage of target DNA or RNA, integration of new spacers (Barrangou, 2013[15]; Wiedenheft et al., 2012[132]). 

#### Cas1 and Cas2: Required in the adaptation process of the CRISPR - Cas system

Cas1 and Cas2 are two proteins in the prokaryotic immune system. Cas1 is an endonuclease that enables the formation of dsDNA fragments. Cas2 is used to buy spacers in the CRISPR system and forms a stable complex with Cas1 and mediates spacer acquisition during CRISPR-Cas adaptive immunity (Wang et al., 2019[128]). 

#### Cas3: Better genome editing tool than Cas9

Another Cas protein is Cas3, and this Cas protein participates in the CRISPR intervention in the third stage of CRISPR immunity (He et al., 2020[45]) and is essential for phage defense in the CRISPR-Cas system (Jackson et al., 2014[57]). While scientific research on the CRISPR-Cas9 system continues, the importance of Cas proteins continues to be discovered. In a new gene-editing method based on Cas3, involving CRISPR-Cas3 and human embryonic stem cell line in HAP 1 (Huntington Associated Protein 1), base sequences in DNA were deleted rather than cleavage (~100 kilobases (kb)). Thus, some technical problems were overcome using Cas9. CRISPR-Cas3 enables the detection of non-coding gene locations in the genome by screening gene elements that are effective in cell differentiation, cancer, and protein expression (Dolan et al., 2019[30]). As well as being beneficial, it can cause the deletion of the virulence properties of pathogens due to viral diseases. One advantage of working with Cas3 is that it can successfully correct deletions and insertions in human cells. A study for the Cas3 protein that deletes a large part of the DNA makes this protein different from the others. Furthermore, the use of Cas3 provided more efficient editing than Cas9 without off-target effects. The DMD gene was repaired with Cas3 in induced pluripotent stem cells (Morisaka et al., 2019[92]). Because of this feature, Cas3 is a better alternative than other Cas proteins in its class. Moreover, setting drug targets can have an important potential in the field of disease prevention and agriculture.

#### Cas4: Fast virus destroyer that creates memories of invading viral elements 

The next Cas protein Cas4 has a role of specific integration on CRISPR spacers. Studies to reveal the importance of Cas4 has shown that bacteria cannot create memory when Cas4 is not present (Zhang et al., 2019[141]). Cas4 helps to create memories of invading viral elements, thus protecting the bacterial cell from virus infection. The system quickly finds and destroys the invader virus in virtue of these memories. In the absence of Cas4, the bacteria create an invader memory but cannot preserve these memories. The reason Cas4 cannot preserve a memory is because of short DNA sequences made up of a small number of base pairs that serve as a recognition point for proteins such as PAM (Kieper et al., 2018[63]). This result also shows that choosing a PAM for successful gene editing is crucial. 

#### Cas5-9: Defence response to virus and maintenance of CRISPR repeat elements

The Cas5 and Cas6 core genes are two of the genes encoding proteins specific to each of the CRISPR systems (Haft et al., 2005[43]). When Cas5 is catalytically active, it acts as a replacement for Cas6. When it is not active, it can take place in interference and adaptation phases. Cas6 and 7 proteins, the product of these genes, belong to the repeat-associated mysterious proteins (RAMP) superfamily and have a sequence or structure-specific RNAse activity involved in the processing of pre-crRNA transcripts (Makarova et al., 2011[83]). RAMPs are a class of RNA binding proteins. These proteins play a role in host immunity. They carry a similar sequence in their DNA versus the sequences of viral invaders. These sequences in the host DNA selectively destroy the virus nucleic acid and provide immunity to itself with an RNA-based strategy. RAMP proteins are involved in this defense mechanism (Wang and Li, 2012[129]).

Cas6-9 proteins are involved in the immune systems of various bacteria. These Cas proteins are called cascade-like complex and protect crRNA (Brendel et al., 2014[20]). Cas7 is effective at interference, binds crRNA and it may be effective in RNA-guided RNA cleavage. Cas8 binds DNA and interacts with a crRNA loaded RAMP. Thus, it can take part in both interference and spacer selection (Makarova et al., 2011[83]). 

Today, the most widely used technology is the CRISPR-Cas9 system. Cas9 is a protein that plays an important role in the immune system of certain bacteria and is an RNA-guided DNA endonuclease enzyme. Bacteria use Cas9 to create a memory, interrogate, and distinguish foreign DNA such as bacteriophage or plasmid DNA (Heler et al., 2015[48]). The CRISPR system has two basic factors: the guide RNA and the nuclease. The guide RNA (gRNA) is complementary to the specific DNA sequence. The nuclease is a molecular scissor to separate target DNA. The gRNA directs Cas9 to its target DNA sequence. PAM sequence is needed for Cas9's cutting function. Cas9 recognizes the PAM sequence and creates a double-strand break at the target locus (Askjaer et al., 2014[9]). Cas9 is a molecule that has problems as well as magnificence. Its complementarity is limited and sometimes it can cut non-specific DNA sequences besides the normal cutting function (Lino et al., 2018[77]). This non-specific cutting function can cause serious problems in therapeutic applications and undesirable consequences in experimental interventions. Besides, its large size makes it difficult to enter cells containing viral vectors (Xu et al., 2019[137]). Finally, the rules required in the PAM sequence limit the manipulation of the target DNA (Gleditzsch et al., 2019[39]). Which is the correct Cas9? At this point, Cas9 variants and alternatives offer various solutions. The most popular Cas variant is isolated from the bacterium *Streptococcus pyogenes* (SpCas9) (Le Rhun et al., 2019[73]). The other Cas types are respectively dead Cas9 (dCAs9), *Staphylococcus aureus* Cas9 (SaCas9), *Streptococcus thermophilus* Cas9 (StCAs9), *Neisseria meningitides* Cas9 (NmCAs9), *Francisella novicida* Cas9 (NvCas9) (Cebrian-Serrano and Davies, 2017[22]). What makes them different is the variety of PAM sequences they need and some are small enough to be easily packaged into viral vectors, such as in SaCas9 (less than 1 kb) (Tsang, 2017[123]). Two new Cas9 variants have been discovered, recently different from known systems: CasX and CasY. CasX, which consists of only 980 amino acids and is found in bacteria not included in the human microbiome. CasX is similar to Cas9 but a very small protein. CasX can be advantageous when our goal is to send small parts to the cell. Similar to other Cas enzymes, CasX can cut dsDNA accompanied by sgRNA, bind to DNA for gene control, and target specific DNA sequences. But it has its unique RNA that performed the same functions (Burstein et al., 2017[21]). The CRISPR-CasY system was first discovered in organisms with a small genome, called Candidate Phyla Radiation (CPR), that rarely encoded CRISPR-Cas systems for phage defense and contains a large bacterial group (Chen et al., 2019[24]). They are few in a number, and little information is available on their function. CasY is also known as CS12d. Cas12d is the class II effector protein. It binds to dsDNA targets and cleaves these targets. Unlike CasX, other CRISPR-based editors are larger and come from bacteria that infect humans. Another remarkable feature of CasX besides its small size is that it is found in bacteria that humans have never been exposed to. Is this an advantage or a disadvantage for genome editing? Cas9 proteins used for genome editing originate from bacteria in the human body and carry an immunity memory for Cas9. For Cas9, it is not clear what will happen about the behavior of the immune system in a person with this memory. Can it be preferable to include CasX and CasY in non-human bacteria for gene therapies to be made in humans? They are questions that researchers need to find answers to. 

#### Cas10 and Cas11: DNA binding, endodeoxyribonuclease activity

The CRISPR-Cas10 system differs from other CRISPR systems because it does not require a PAM sequence and can identify sequences even in the presence of point mutations (Bari et al., 2017[14]). CRISPR-Cas10 system is a potential candidate for gene editing due to these features. Cas11 is a subunit in type I and III (Dorsey et al., 2019[31]; Majumdar and Terns, 2019[82]; Shmakov et al., 2018[112]) effector complex and involves in the maintenance of CRISPR repeat elements (Majumdar and Terns, 2019[82]). There are a limited number of studies about the basic functions of Cas11. Classification in the CRISPR-Cas system includes effector modules in class 1 systems. These modules consist of multiple Cas proteins. Some of these are in the form of crRNA-binding complexes (cascade complex 1), and this form mediates pre-crRNA processing and interaction with the contribution of additional Cas proteins. As described herein, Cas11 is a member of effector modules and is involved in crRNA processing and interference. The interference or effector module is involved in target recognition and nucleic acid cleavage. Additionally, the effector modules have different combinations of Cas protein (Shmakov et al., 2018[112]). The purpose of these modules is to target surveillance and defense against foreign genetic material as viral genomes. In other words, this suggests that Cas proteins may play a role in the uptake of new spacers, as well as their effects on the combination of the effector complex, depending on the type of Cas in the CRISPR-Cas system.

#### Cas12 and Cas13: Affect gene expression without changing the genome sequence for therapeutic application

Two of the most studied Cas proteins after Cas9 are 12 (Cpf1-Cas12a) and 13 (Swarts and Jinek, 2018[119]). Cas12 is a class 2, type V effector protein (Makarova et al., 2020[85]), and is an effective enzyme that produces cascade cleavage in dsDNA. It is used as a platform for the regulation of the epigenome as it can process its guide RNA. It is also known that Cas12a can arbitrarily cleave single-stranded DNA after it has been activated by a target DNA molecule that matches the spacer sequence (Chen et al., 2018[23]). In this way, Cas12a can detect a small amount of DNA in a mixture and uses an RNA molecule as a guide to find the complementary DNA sequence. Cas12 cuts both strands of the target DNA it determines (Rusk, 2019[107]). The cutting process initiates the repair process that causes changes or editing in the genomic DNA sequence (Yao et al., 2018[138]). Cas proteins targeting RNA are promising for the antiviral approach in treatment. Cas13 is a class II and type VI protein (Terns, 2018[120]). It effectively targets and cleaves of RNA in mammalian cells (Abudayyeh et al., 2017[2]) and various model systems (Pickar-Oliver and Gersbach, 2019[101]). Today, effective and cost-effective solutions are needed for the diagnosis of diseases. Synthetic biomolecules are used as a solution. However, it may not be possible to provide all of their specificities, sensitivity, speed, cost and convenience together, but it may not be possible to provide all their specificity, sensitivity, speed, cost, and convenience together. Recently, a Specific High-Sensitivity Enzymatic Reporter UnLOCKing (SHERLOCK) complex was created with an RNA-guided and RNA-targeted CRISPR-Cas13a. SHERLOCK has high sensitivity and specificity and is very suitable for detecting target RNA and is a CRISPR based diagnostic. However, it is unsuitable for detecting DNA sequences, and *in vitro* transcription of DNA to RNA must be performed before the SHERLOCK test. In the study conducted to solve this problem, CAS12a/crRN/ target DNA triple complex was created. By adding a quenched fluorescent ssDNA reporter to this system, an Hour Low-Cost Multipurpose High-Efficiency System (HOLMES) was developed. The system can be used for the rapid detection of target DNA as well as target RNA (Li et al., 2018[74]). It should not be overlooked that the CRISPR system has deficiencies as well as its benefits. As with other diagnostics, this system is generally not perfect. However, its applicability is easier and faster than previous gene therapy systems. It is a great advantage that the system has a high capacity to accept accessory molecules and can be shaped according to the treatment goal.

Cas13 has CRISPR array processing efficiency and is ideal for multi-target applications. Most importantly, it is widely used in biotechnology and clinical applications. It is possible to specifically identify targeted DNA or RNA sequences using Cas13 (SHERLOCK) (Kellner et al., 2019[62]). The clinical significance of this system is that RNA or DNA can be detected multiplexed and sensitively in relevant samples. When we look at the features of this system, we see that we can target mammalian viruses and thus create an antiviral platform. This platform allows investigation of the prevalence of target regions in viral genomes containing ssRNA and thus enables the identification of possible targets. However, there is still a need to develop strategies to determine whether the Cas13 activity is being performed correctly and to optimize this behavior while scanning these targets. The SHERLOCK system has been used in some patient samples to detect different viruses (Myhrvold et al., 2018[94]). The system searches for specific nucleic acids, and test strips are used to make them visible. The presence of the virus is determined by the line that will be formed after the paper strip is dipped into the sample to be examined. Using these systems, researchers are working on CRISPR-based systems such as Ebola, Zika, Lassa (Myhrvold et al., 2018[94]) and COVID-19 (Bai et al., 2020[11]) that allow detection in the event of an epidemia. The results are promising. Because instead of using the CRISPR application that requires training, this method enables laboratory personnel to obtain viruses directly from biological samples such as blood and saliva and to conclude them quickly. In this way, cost, time and equipment is saved. Cas13 is the only known prokaryotic CRISPR-Cas system that targets the single RNA. Therefore, RNA has more places in specific applications and is preferred over other Cas types for targeted RNA degradation and gene knockdown, RNA editing, nucleic acid detection, and patient diagnosis (Terns, 2018[120]). 

#### Cas14: Benefit and loss accounting compared to Cas9, 12 and 13

Cas14 is also advantageous in biotechnological applications. Cas14 is an RNA-guided nuclease and can provide targeted ssDNA cleavage without requiring restrictive sequences. An important detail is that Cas14 does not require a PAM sequence and recognizes ssDNA more specifically than Cas12 and 13. Cas14 is a newly discovered Cas protein. Although Cas14 is smaller than Cas9, it can target ssDNA in defense against viruses with the ssDNA genome. Owing to this feature, Doudna and her team combined Cas14 with the isothermal amplification method called DNA endonuclease-targeted CRISPR trans reporter (DETECTR-Cas14) (Harrington et al., 2018[44]). Cas14 has a big advantage due to its small size. Cas14 can provide regulation in both cells and viral genes and contribute to the development of CRISPR-based diagnostic systems when it comes to infectious diseases, mutations, and cancer with ssDNA cutting activity. Cas9, 12, 13 and 14 are in a competition that can vary depending on the single or dsDNA or RNA they target. It would be wrong to say which of these is the best. Cas12 is successful in recognition of dsDNA, recognition of Cas13 ssRNA and finally recognition of ssDNA of Cas14. It is understood that Cas's alone are enough to fulfill their functions. Can SHERLOCK and DETECTR be combined? These techniques are diagnostic tools that can be used to specifically detect low concentrations of RNA and DNA. Heating Unextracted Diagnostic Samples to Obliterate Nuclease (HUDSON) technique is used to directly detect nucleotides in body fluids. When HUDSON and SHERLOCK are used together, they can be combined to detect RNA and DNA in a small amount of sample urine, saliva, serum, plasma and whole blood (Gronowski, 2018[40]). This system allows the editing of mutations and genes, rapid identification of viral and bacterial pathogens, and pathogen-resistant genes in the same analysis. Aiming to increase the target specification of CRISPR-Cas9, researchers focused on Cas9, 12, 13 and 14. These 3 proteins have different advantages as diagnostic. Cas proteins must meet certain criteria. Also, the character of the nucleic acid sequence the user wants to detect is important. Specific nucleic acid sequences to be detected are cut with protein guide molecule complexes. The advantage of these complexes is that they do not specifically cut other nucleic acids after the target sequence has been cut. When the cutting process is finished, they generate a visual signal along with dCas9 labeled with an enhanced green fluorescent protein (eGFP). Only user-specified nucleic acids are truncated (Zhou et al., 2018[143]). The aim is a fast and reliable diagnostic combination. The key point of these systems is the type of Cas to be used. The advantages or disadvantages of the system, depending on the Cas's, depending on the length of the protein, the size of the single guide molecule or the accuracy of the process and the nature of the target to be manipulated.

The main criteria to be considered in the use of the system include the type of Cas protein and system to be used (HOLMES, SHERLOCK, HUDSON-SHERLOCK, DETECTR, SHERLOCK-DETECTR), effector molecule, sensitivity, specificity, fluorescent molecule, type of the target (DNA, RNA, protein) and duration (Kostyusheva et al., 2020[69]; Li et al., 2019[75]).

## Crispr Behavior in Biological Systems

Researchers have focused on solving current problems such as human diseases, food quality and productivity by developing CRISPR technologies. Human diseases take the first place among the purposes of CRISPR applications. The most easily treatable group is mutational diseases with a direct relationship between the cause of the disease and the clinic. One way of treating diseases using these methods is to mimic the disease by modeling it in cell, model organisms, or transgenic animals, due to ethical concerns in human studies. In addition, they can be implanted into cells by plasmid transfection to model cellular defense in infectious diseases and can be used to create disease models at the cellular level. With these techniques, we can edit the DNA of organisms, delete genes at any stage of its formation, and even the entire chromosome. This deletion procedure can be useful in the treatment of extrachromosomal disorders such as Down syndrome and Triple X (Cowan et al., 2019[26]). Today, CRISPR-Cas technology is used in diseases such as cystic fibrosis, hemophilia, DMD, sickle-cell anemia, β-thalassemia, cancer, and Huntington's disease. Research continues for MHC proteins, which are important for tissue engineering and stem cell engineering, regenerative medicine (Hsu et al., 2019[53]), and tissue and organ rejection (Abrahimi et al., 2016[1]). These initiatives predict that CRISPR will find more places in our lives in the future. As such, these technologies seem important not only in gene editing but also in synthetic biology. The purpose of synthetic biology is to create bio-computers, to produce genetic circuits that work and have memory. Thus, it is planned to program mammalian cells using Cas9 variants, which are protein-based processors that control gene expression in response to single-cell RNAs (Kim et al., 2019[64]). Is it possible to record on DNA using this data? To store the data in DNA, the researchers created an empty recording space that mimics the "0" and "1" system in the computer. Then, based on the 4 bases in DNA, they evaluated "A and C" as 0, "T and G" as 1, according to the Watson-Crick pairing, and found that these data were correctly translated by the system. As a result, this system was envisioned as a natural mechanism by which a copy of DNA is created (Church et al., 2012[25]). With the CRISPR system, it may be possible to store data in DNA. The creation of this code, which will be reflected in the entire living system, may have critical consequences for the organism. However, it seems possible and controllable to create this software in a single cell. Recently, the CRISPR gene editing system was applied for the first time in one patient. In this procedure, white blood cells of two patients with multiple myeloma and sarcoma were obtained. These cells were arranged with the CRISPR system and reintroduced to the patient (Stadtmauer et al., 2020[114]). To develop antitumor immunity in patients' T lymphocytes, the CRISPR technique was applied to TRAC, TRBC, and PDCD1 genes and New York esophageal squamous-cell carcinoma 1 (NY-ESO-1) was used to recognize tumor cells as well-known cancer-testicular antigen (Thomas et al., 2018[121]). As a result, it was observed that patients could tolerate this gene therapy. The results regarding the long-term effectiveness of the application, the patient's life and recovery time, or the relapse of the disease are closely related to the individual responses of the patients, and the success of the application depends on many factors. Treatment approaches are promising. What would happen if this change was passed on to future generations? Gene drivers created using the CRISPR system makes it possible to design certain features that can be transferred between generations. For this purpose, the allelic drive system equipped with guide RNA, which allows replacing the unwanted variant of a gene with its preferred variation, has been developed. Desired genetic features such as drought resistance, increased crop yield, resistance to pests, sensitivity to pesticides can be spread to populations or products can be given resistance to mutagens. This study shows that insects are a good source for allele drivers (Guichard et al., 2019[42]). By adding the engineered gRNA to a gene driver, it was found that the allele gene driver spreads through the population and repairs the sensitive allele by encountering the resistant allele. The most important observation of the study is that errors caused by allelic drivers are not transferred to the next generation and as a thought-provoking result, an extraordinary situation called "fatal mosaic" emerged. It is thought that this can be used as an advantage to eliminate unwanted mutations caused by drivers containing CRISPR and make them more efficient. Based on this application, in order to correct the faulty allele with the CRISPR-Cas9 system in human diseases, this faulty allele must be enzymatically cut and corrected by replicating the error with an exogenous oligonucleotide or dsDNA template. In a study on this subject, it is aimed to show that it is possible to repair the other allele to be sensitive to cutting by creating a cut-resistant allele for heterozygous individuals in the germ line (Guichard et al., 2019[42]). This model can be applied in animal models and cell lines to mimic many human diseases. Although allelic correction and intergenerational transfer seem to be good practice, as stated before, unexpected results may be encountered due to the uncontrollable features of the CRISPR-Cas9 system. Another study on this subject is the deletion of the memory of rats with CRISPR (Sun et al., 2020[117]). This important claim will pave the way for the CRISPR system, to which we refer to rewritable codes, to erase and restore memory and perhaps load data that will at least restore people's daily routines in diseases such as Alzheimer's. However, considering that memory is not alone and epigenetic memory is effective in emotions, situations, and behaviors, it is still uncertain how the transfer of an epigenetic molecule pool to individuals with CRISPR intervention will have consequences. At this point, organisms that inspire genetics are very important. Understanding these organisms enables the determination of treatment options in humans by creating appropriate models. As a fundamental mechanism, a major change in DNA is transmitted by the mRNA from the nucleus to the cytoplasm. If RNAs are arranged outside of the nucleus, what would be the result? The answer is given by a group of researchers on the *Doryteuthis pealeii* (longfin inshore squid). Researchers reported that this cephalopod was the first known animal to regulate messenger RNA outside the cell nucleus (Vallecillo-Viejo et al., 2020[124]). Permanent changes in DNA make rearrangement difficult to do here. However, when using mRNA, it is possible to avoid treatment-induced errors due to its half-life. When an RNA editing similar to that of squid is made in the cytoplasm, if the result is outside what is expected, the regulation can be done by replacing it with an analog. In this way, cytoplasmic correction of mutations will pave the way for personal treatment in humans. Editing between the nucleus and the mitochondrial genome, can be fatal to mitochondria due to the lack of a mitochondrial repair mechanism. The sequence of the mRNAs, the proteins they encode and the information they carry can be changed by intervening in the plan of the genome without disrupting them. To this end, the researchers demonstrated that genetic information can be re-encoded in a region-specific manner in the regulation of neuron RNA via the ADAR2 enzyme, which is expressed outside the nucleus of squid neurons. In mammals, this enzyme is both diverse and largely expressed in the nucleus and nucleolus. This mechanism in squid can be mimicked by evaluating its molecular structure, diversity and cellular location and can be applied in human cells and animal models. It is stated that such a control can be achieved with small-molecule inhibitors of *Streptococcus pyogenes* Cas9 (SpCas9) protein (Maji et al., 2019[81]). The biggest concern in this application is in reducing the off-target effects of Cas9. In solving this problem, it may be useful to design and use small-molecule inhibitors (anti-CRISPR) for CRISPR-related nuclease using computational biology. The problem with existing anti-CRISPR molecules is that they are large and cannot enter the cell. Small-molecule inhibitors are favorable because they are recyclable and are not degraded by cellular proteases and immune system reactions. 

The cellular security provided CRISPR system can be used to turn genes on or off. This initiative could allow genes to be managed when, where and for how long. In a new method on this topic, CRISPR gene editing enzymes were injected directly into the brain of a developing mouse fetus as a therapeutic intervention (https://www.spectrumnews.org/news/injecting-crispr-fetal-brain-may-correct-autism-mutations/). The study is based on the opening of the UBE3A gene before birth, which causes Angelman syndrome as a result of a mutation in the developing brain. The results of the study conducted in 10 mice show that the gene reactivated using CRISPR at the age of five months after treatment works in about half of the neurons in the brains of mice. As in these studies, genes can be silenced or activated with CRISPR technology. The application of the procedure at the developmental or stem cell stage is sensitive, and it is important to which part of the animal it will be performed (tail, head or brain). Another interesting CRISPR application was discovered by researchers working on pain. The subject of the study is an antidote containing CRISPR technology that reduces pain caused by Australian venomous jellyfish venom (Lau et al., 2019[72]). The genome editing technique used for this deadly venom can improve symptoms 15 minutes after exposure. Can pain be treated with this method in human pain panel and chronic diseases? There is a wide spectrum from migraine to rheumatic pains. The technology subject to the research was applied by the injection method. The aim of the researchers is to develop the form that can be applied to the skin. At this point, the problem we always face is that CRISPR has off-target effects by cutting the wrong piece of DNA. The technology applied is similar to the use of stem-loop primers in *in vitro* analysis of short sequence miRNAs. In the method, in addition to the CRISPR mechanism, a short tail of 20 nucleotides locked to the gRNA was added. When the tail reaches the target RNA, it folds back and binds to the RNA, forming a hairpin that allows it to cut Cas9 exactly at that site. As new molecules and applications were discovered, tests performed on different CRISPR systems involving human cells show that gene editing accuracy has increased (Kocak et al., 2019[65]). Can we solve every problem we encounter with CRISPR? Looking at the researchers, publications and the presence of numerous opened research companies, especially the recent COVID outbreaks have led us to turn to this technology, which has the most common and easily applicable potential. As a result, the CRISPR-Cas system is an effective and promising technology for the today and future.

### LEAPER

The changes in the nature of CRISPR technology have led to the search for new alternatives. One of these changes is the development of LEAPER technology, which is thought to be safer and has fewer side effects by targeting RNA instead of DNA (Qu et al., 2019[103]). This technique includes endogenous ADAR (ADAR1 and ADAR2) and native proteins. Thus, immunogenic reactions can be prevented. The technique requires the use of specially engineered RNAs (aRNAs-ADAR RNAs) that combine naturally-occurring enzymes to convert specific adenosine to inosine. While this application is successful in cellular studies, research continues in rats. Recently, a cytidine and adenosine deaminase has been used to correct disease-causing mutations. In this method, DNA base editors were constructed, and ADAR adenosine deaminases were used to precisely regulate RNAs to adenosine-inosine (A-to-I) and combined with CRISPR-Cas9 (Qu et al., 2019[103]). A-I editors have a broad spectrum and can affect major mechanisms that can affect gene expression, such as modification of protein-coding sequences, splice site modification, RNA's ability to modify itself against nuclear exposures, microRNA sequences, and their target regions (Quinones-Valdez et al., 2019[104]). Three types of ADAR proteins have been identified, the substrate of which is a double chain RNAs. During translation, inosine is thought to mimic guanosine (Shevchenko and Morris, 2018[109]). This change is important. Because many of the known diseases can be corrected using this change. Hurler syndrome is one of them and a rare lysosomal storage disease. To treat this syndrome, a ZFN-mediated gene editing study was performed using the murine model, and a correct copy of the IDUA gene was inserted at the albumin locus in hepatocytes. Thus, this metabolic disease was corrected by providing continuous expression of the enzyme (Ou et al., 2019[99]). As shown by this study, LEAPER is preferred in similar studies because it is simpler than existing techniques. The distinguishing feature of LEAPER from other techniques is that CRISPR requires components such as gRNA and Cas enzyme, whereas the only aRNA is used in this technique. Thus, a gene-editing alternative is provided with low immune rejection and easy delivery to the target. In this study, it is aimed to restore the catalytic activity of α-1-iduronidase (IDUA) in the primary fibroblasts of patients. The promises of technologies to correct genetic errors are striking, but the problems of these technologies cannot always be tolerated by the editing system or the elements in the system. Abnormal regulation of RNAs in multiple myeloma where ADAR1 is overexpressed leads to off-target regulation by increasing oncogenicity. Moreover, ectopic expression of proteins is a potential risk for immunogenicity. Because the adaptive immunity in the cell and the DNA repair response involving p53 may compromise the efficacy of a therapeutic protein such as Cas9. However, some studies favor ADAR because it does not have immune-stimulating effects and does not interfere with the function of endogenous ADAR proteins. When engineered ADARs for RNA editing are examined, apart from the A-to-I editors mentioned so far, there are also C-to-U editors, although they are not often mentioned (Fry et al., 2020[35]). A deaminase has been developed that allows the regulation of cytosine and uracil in RNA by mutagenesis of ADAR2. The disease in which ADAR technology is applied is hereditary retinal degeneration. In this disease, blindness occurs as a result of the death and dysfunction of outer retinal cells due to mutations in the heterogeneous gene sequence. Researchers hope that this treatment could stop or reverse vision loss in patients. LEAPER is preferred because it is a target-specific, reversible technology that does not have low immune reactions, does not have the risk of creating permanent non-target mutations. Also, LEAPER technology has been used to correct pathogenic G>A change in cells (Abudayyeh et al., 2019[3]). One of the therapeutic targets of LEAPER is the tumor suppressor gene TP53 (non-functional truncated protein is formed) (Floquet et al., 2011[33]). The gene is primarily responsible for maintaining cellular homeostasis, and the frequency of mutations in the gene is high in more than 50 % of human cancers. This study demonstrated that LEAPER repairs the early stop codon of TP53 and restores its function. The ability of aRNAs to be efficiently delivered to the cell as vectors or as a synthetic oligonucleotide is promising for therapeutic applications with high regulatory activity. The application spectrum of this technology is wide. One of these is miRNAs and they are a good resource because of their therapeutic potential. MiRNAs have multiple targets, and multiple miRNAs are specific to a single target. In addition to these features, the seed sequences of miRNAs are suitable for editing as they allow A to I regulation. miRNAs are translationally repressors and affect gene expression. Given that many genes are regulated by miRNAs, mapping hotspots for editing can be a guide for treatments such as cancer (Wang et al., 2017[130]). With this technology, diseases can be treated by changing the main targets of irregular miRNAs (van der Kwast et al., 2018[125]). Regulation in the miRNA seed region determines mRNA selection and gene silencing activity (Kume et al., 2014[70]). Moreover, this regulation allows interfering with RNA metabolism, involving the folding, processing, localization, and degradation of RNA (Hong et al., 2015[51]). Since RNA folding also affects the biology of the molecule, the folding pattern and secondary structure properties can be revealed using databases. Using these databases, it contains folding information for each base pair, and the folding properties of molecular elements such as metric computation, coding, non-coding and intergenic regions, repeating elements, telomeres, and transposons can be calculated. This computation can help us analyze RNA folds with LEAPER technology and discover the abilities of regulatory elements and non-coding RNA (Andrews et al., 2017[5]). Because RNA folding errors must be known to be able to edit. A study reveals the importance of this idea. The study demonstrates the complex and regulatory role of ADAR1, which forms the basis of LEAPER technology, on RNA secondary structure (Solomon et al., 2017[113]). Errors due to re-expansion of microsatellites due to RNA secondary structure are generally seen as myotonic dystrophy types 1 and 2, Fragile-X, amyotrophic lateral sclerosis, frontotemporal dementia, and tautopathies (Bernat and Disney, 2015[17]). If A-I editing is done, the structure and base pairing property of the RNA molecule may change. This change is important in controlling the number of functional RNA molecules in the cell and providing cell defense (Wulff and Nishikura, 2010[136]). A-I editing can be used as a system with RNA basic regulators consisting of Cas13 and ADAR. Thus, higher editing efficiency can be achieved by targeting more specific. It is important to consider ethical rules in researches on human and other living life. Before genome editing becomes an intervention with unpredictable results, initiatives that can control individual responses and reduce ethical concerns should be included, especially in human applications.

### RESTORE

Today, the aim of gene editing technologies is to restore hotspots needed in the genetic basis of organisms. RNA editing can be a reliable and effective alternative to gene editing in clinically specific situations. Another application of ADAR is based on the control of exogenous RNA-regulating enzymes or overexpressed endogenous ADAR enzymes. This method involves the use of chemically optimized antisense oligonucleotides that call ADARs to the task of editing endogenous transcripts (Merkle et al., 2019[89]). The technique is a successful practice where off-target regulation is almost non-existent and natural balance is not disturbed. This technique has been applied in standard human cell lines and human primary cells to treat Alpha-1 antitrypsin (alpha-AT) deficiency, which is among the most common genetic causes of liver disease in children. 10 % of affected patients have homozygous alpha-ATZ mutation (PIZZ) (Mencin et al., 2007[88]). Using the RESTORE technique, the PIZZ mutation was repaired by administering a single oligonucleotide to eliminate the ectopic inhibition of proteins in patients. The study aims to use chemically stabilized ASOs (RESTORE) instead of plasmid-induced gRNA expression to increase the efficiency of RNA rearrangement. While designing ASOs, natural ribonucleotides, chemically modified domain of specificity (2′-O-methylation, phosphorothioate) and ADAR was used. As with other gene-editing technologies, the main goal in this technology is to prevent immune rejection. Therefore, the regulation of genes in cells taken from volunteers and the reintroduction of these altered cells by planning to produce the missing protein or attack the faulty protein may be preferred in terms of preventing immune rejection reactions. The basis of the process is endogenous ADAR uptake into specific transcripts for oligonucleotide-oriented RNA editing. Several techniques have been tried in different combinations in the study. In this study, various techniques were tried in different combinations. First, 15 different sequences designed to identify sites that collect the best ADAR were identified, and it was concluded that the use of ADAR1 may be useful for RNA editing. Second, antisense oligonucleotides targeting the 5 'UAG sequence in the 3'-UTR region of ACTB or GADPH, which are often used as endogenous controls in gene expression studies, has been designed. Thus, it was determined, which antisense oligonucleotides preferred ADAR. Finally, the simultaneous regulation of both transcripts by transfection of two antisense oligonucleotides was investigated. Editing effectiveness was found almost the same. For this region, it is noteworthy that the RNA editing for this region may be performed in several transcripts at the same time. Antisense oligonucleotides can be an alternative for plasmid-derived gRNAs. This method applied to ADAR enzymes may be possible in other enzyme systems in the future.

### ARCUT

Homologous recombination is a way of changing the genome in a certain way. It is a rare event in the cell, and its frequency can be increased by creating a double thread break in the target area. Technologies such as HE and ZFNs require that this area be recognized by the molecules in the system when the target sequences change. Therefore, their application takes a long time and is laborious. ARCUT technique has been developed to overcome this problem. In this technique, a cutting and artificial oligonucleotide derivative is created and specifically recognizes the target sequence, although it is long, as it can be designed to correspond to the target gene (Shigi and Komiyama, 2010[110]). The ARCUT system, designed to support homologous recombination in human cells, consists of molecular scissors called EDTA, ethylenediamine-N,N,N',N'-tetraacetic acid IV (CE (IV) / EDTA) and two peptide nucleic acid sequences. In addition, this system does not contain protein (Katada and Komiyama, 2011[60]). With this technique, the Ce (IV)/EDTA complex is used for the site-specific hydrolysis of ssDNAs, and efficient and selective hydrolysis of the phosphodiester bonds in the spaces formed in the determined positions in the substrate DNA is ensured. The fragments formed in this way were linked with T4 DNA ligase, and recombinant DNAs are produced. Consequently, single-stranded DNA can be manipulated with ARCUT (Sumaoka et al., 2006[116]). Watson-crick rule is used for easy, accurate, effective design, and synthesis of ARCUT (Komiyama, 2013[67]). Specific cutting of the target is a critical step for the system to be used. Induced double-strand breaks are recognized by the cellular repair system and targeted homologous recombination is promoted. Transforming a site to the desired sequence with ARCUT is useful for facilitating the modification of the targeted genome for gene therapy applications by promoting homologous recombination in human cells, and understanding the molecular mechanisms of homologous recombination (Komiyama, 2013[67]). In a study using ARCUT, the telomere lengths of 11q, 12q, and Xp/Yp human chromosomes were compared by trimming the telomeres (Komiyama, 2014[66]; Shigi et al., 2017[111]). The researcher's aim is to detect their effects by clipping telomeres with different telomere lengths in everyone, in each cell of the individual, between the chromosomes and homologous chromosomes in a single cell of the individual, and even between the p and q arm of the same chromosome. In this study, the subtelomeric region of the chromosome was cut and a complementary sequence was synthesized to this cut region. The telomeric repeat sequence is the same in all chromosomes and individuals ((TTAGGG)n). However, the number of repeats is different in each individual, and each chromosome is unique. Telomeric properties are combined with ARCUT, and it is important to reveal many telomeric functions, from aging to preservation of chromosome integrity, both to understand their molecular functioning and to enable them to be used for therapy purposes. As a result, it was found that telomere lengths are significantly different from chromosome to chromosome, and the number of cell divisions is exactly the same, even though they originate from the same cells. Telomeres are seen by researchers as cellular timekeepers that limit a cell's ability to reproduce because they shorten with each cell division. The ARCUT technique can help control these mechanisms, as telomeres are constantly shortened, aging, mutations and cell divisions are terminated by apoptosis, changes in DNA sequences and lead to the programming of cell death. Another feature that makes ARCUT special is that it does not contain enzymes against nonspecific segments caused by some enzymes, and it increases target specificity by facilitating DNA manipulation. With this method, the human genome can be selectively cut from any region. The fragments obtained by this specific cut can provide important information regarding the biological function of the genome. DNA/RNA hybrids are common in human cells and can be selectively hydrolyzed by this method, not just genomic sequences. DNA/RNA hybrids are associated with some human diseases and transcriptional regulatory functions. Hybrids are observed when the newly formed RNA in the cell is close to the DNA chain and is called the R cycle (Ito et al., 2009[56]; Wang et al., 2018[126]). The role of these hybrids in human diseases is thought to create a susceptibility to chromosomal breakage (Nadel et al., 2015[95]) and Wiskott-Aldrich syndrome (Sarkar et al., 2018[108]), an immune-defective disease such as Friedreich's ataxia (Neil et al., 2018[97]), Amyotrophic Lateral Sclerosis 4 (ALS4) with a neurodegenerative disease and senataxin mutation (Grunseich et al., 2018[41]). Detecting or sequencing these hybrids in the genome are important both for understanding transcriptional regulation and for interfering with these targets. DNA/RNA hybrids are not alone in doing their job. When the proteins associated with these hybrids were examined, it was seen that they were involved in gene silencing due to methylation. It may be useful to use ARCUT technology in gene silencing, which is one of the purposes of gene editing technologies. ARCUT technology is as useful as it has problems to solve. The researchers suggest that this technique can be made more flexible and versatile to address its inadequacy in difficult targets such as GC-rich regions. The system is not effective in environments with high salt concentrations. Cutting the duplexes targeted by ARCUT in the cell with abundant metal ions poses a problem in terms of specificity and efficiency. Finally, *in vivo* applications, it incisors should be transferred to the nucleus and localized where the target to be cut is located (Komiyama, 2014[66]). When the deficiencies of the ARCUT technique are completed, it may be possible to manipulate the genetic material of all organisms by selectively cutting.

### SATI

A large part of the mutations responsible for genetic diseases occurs in non-coding regions of the genome. Non-coding regions are involved in gene regulation, even though they are not directly involved in protein. These regions are responsible for cell functions such as turning genes on and off. These properties make them advantageous for gene-editing technologies. Repair of non-coding region mutations can provide many advantages for the cell. The SATI technique has been considered as an alternative for these mutations.

The cell-linearized Single Homology Arm donor-mediated intron Targeting Integration (SATI) technique uses two DNA repair mechanisms known as HDR and HNEJ. The purpose of SATI is to take advantage of one of these repair mechanisms and insert a new segment to genome without removing the old fragment. The advantage of the technique is that while unpredictable results occur in protein-coding regions, it does not pose a risk for unwanted or deadly consequences due to the non-coding region. CRISPR is very effective in dividing cells such as skin and intestine. Because it takes advantage of the cell's repair mechanisms. The goal of the researchers in developing homology-independent targeted insertion (HITI) modified from CRISPR is to target both dividing and non-dividing cells (Suzuki et al., 2019[118]). HITI uses the NHEJ repair path that targets DSBs. With the SATI technique, DNA is knocked in using HITI, thereby targeting many mutations and different cells. The researchers managed to increase the survival time of mice with the SATI technique in mouse models they created for the treatment of progeria. In addition, observation of improvement in multi-organ systems has shown that the treatment is effective. In this study, the protein was returned to normal as a result of replacing the defective copy that caused the disease with a normal copy. Also, it is possible to coordinate the operation of the system by adding any enhancers or insulators, and it can be an advantage to monitor the cell line in non-dividing cells. By interfering with stem cells, it may even be possible to change the fate of the cell, and it can be used to repair mutations, repair the recessive mutant allele and produce functional proteins.

### RESCUE

RNA regulation (RESCUE) for specific C-to-U exchange is the RNA regulation method created by serial mutagenesis (Fry et al., 2020[35]) of fused ADAR deaminase domains (ADARDD) to dRanCas13b (Cox et al., 2017[27]). The need for new techniques is due to deficiencies in existing technologies. CRISPR is the most preferred, applied, and even tried method by combining it with different methods. However, it is the disadvantage of the technique that CRISPR creates permanent changes. When the editing of bases is done by targeting RNA, the genome allows for temporary editing. Current technologies are limited use because they target a type of mutation. The RESCUE technique developed to solve these problems is both CRISPR based and aimed at a wider target. Editing of RNA by changing bases is short-lived and can be used for some treatments without causing a permanent change in the genome, basically edits owned sequences by cutting them and can cause an off-target effect. RESCUE is directed towards a single target and reduces the likelihood of unwanted changes in cells. The RESCUE system uses an enzyme that can change C to U and expands the application capacity of CRISPR. The researchers believe that the natural enzymes used in this technology that catalyze the C-to-U conversion can exhibit off-target behavior because they only work on one strand, and have multiple deaminations, even if they are used in editing in DNA (Cox et al., 2017[27]). Therefore, they developed a technique that is effective in dsRNA substrates and targets C-U exchange. By activating β-catenin in mammalian cells, they combined cytidine deaminase and dCas13 to regulate cell growth and increased the specificity of RESCUE with rational mutagenesis. As a result, they obtained a high specification for C-to-U editing tool. The width of the amino acid codon domain in RESCUE technology allows for modifications such as phosphorylation, glycosylation, and methylation, and targeting catalytic residues, mutations in diseases and protective alleles such as ApoE2 (Abudayyeh et al., 2019[3]). It is a technology that can be a solution in diseases as obesity (ApoB RNA editing) (Blanc and Davidson, 2008[18]), Alzheimer (ApoE2 RNA editing) (Wu and Zhao, 2016[135]), and cancer (especially Breast cancer-APOBEC3 RNA editing) (Asaoka et al., 2019[8]) within the framework of these mechanisms. RESCUE is a new technology and there are technical problems to be solved. It has an infrastructure that can be developed and can be combined with existing technologies.

Although all genome editing technologies have different advantages over each other, they also have disadvantages (Table 2(Tab. 2); References in Table 2: Agrotis and Ketteler, 2015[4]; Aquino-Jarquin, 2020[7]; Bayat et al., 2017[16]; Botella, 2019[19]; Fry et al., 2020[35]; Gronowski, 2018[40]; Grunseich et al., 2018[41]; Harrington et al., 2018[44]; Islam and Lai, 2019[55]; Ito et al., 2009[56]; Katalani et al., 2020[61]; Kellner et al., 2019[62]; Li et al., 2018[74]; Li et al., 2019[75]; Lin et al., 2014[76]; Nadel et al., 2015[95]; Neil et al., 2018[97]; Ou et al., 2019[99]; Sarkar et al., 2018[108]; Suzuki et al., 2019[118]; Wang et al., 2018[126]; Wu and Zhao, 2016[135]) and there are still gaps to be re-solved. Life cycles of organisms are subject to dynamic processes, and many factors such as environmental interaction, genetic infrastructure, changing living conditions have a say in biochemical processes and mechanisms.

## Conclusions and Future Perspectives

The vast majority of the studies conducted so far are on somatic cells. Somatic genome editing involves altering genetic resources to completely cure the disease due to a genetic mutation. The cell to be changed is taken from the individual and the relevant mutation is corrected with an appropriate genome editing technology and given back to the patient. With treatment, cells are altered, but germ cells are not affected. Interference in germ cells can affect each cell and subsequent lineages. However, cell culture and animal experiments can be useful in observing functioning and future results. The situation is more serious when it comes to embryo intervention. Studies still have deficiencies for embryo intervention. While researchers have concerns about IVF treatment, today many individuals can have children with this method. Considering that the treatment of single mutation diseases is possible with genome editing methods, it may be the last point to achieve this in the embryo. Perhaps the effects of genome editing can be demonstrated using twin births in animal models. In terms of controllability of genome editing results, alteration or deletion of a gene may cause off-target effects, and clinical results may not be predicted. For example; Embryonal genome editing for the viral source of infection may pose a risk in individual response to other sources of infection.

Until today, gene editing principles in model organisms have been investigated, and their homology from humans has been sought. By going back to the essence of the human genome, the search for proteins that work similarly to Cas, even smaller and serving as enzymes, may provide convenience in terms of the safety of therapies that will not cause immune rejection, that is, use self-propelled therapies. 

The biggest challenge in genome editing arises from the target arrival and retention time of the created components. If we lean towards protein targets rather than genes, we can create a system that does not need to be constantly expressed and can degrade rapidly, and off-target effects can be reduced. Thus, healthy and productive plants and animals can be obtained with proteins that are not integrated into the genome.

The efficacy of the method and the specificity of the target are promising for many diseases, and it can assist not only in the treatment but also in understanding the dynamics of the genome. With these systems, by editing the human genome, it may be possible to prevent, slow down or extend human life due to systemic and mental aging. All methods are in the form of intertwining technologies. The suitability of the method depends largely on the target to be investigated, technical possibilities and the nature of the error to be corrected. There are still open points in genome editing technologies in terms of efficiency and specificity. Shaping epigenetic memory, methylation, histone packing, repair mechanisms, crossing-over, sibling chromatid exchange, reverse genetics, advanced genetics and many other genome mechanisms in organisms that can be used for the human and human benefit are waiting to be explained and intervened in their behavior in disease and health.

## Figures and Tables

**Table 1:**
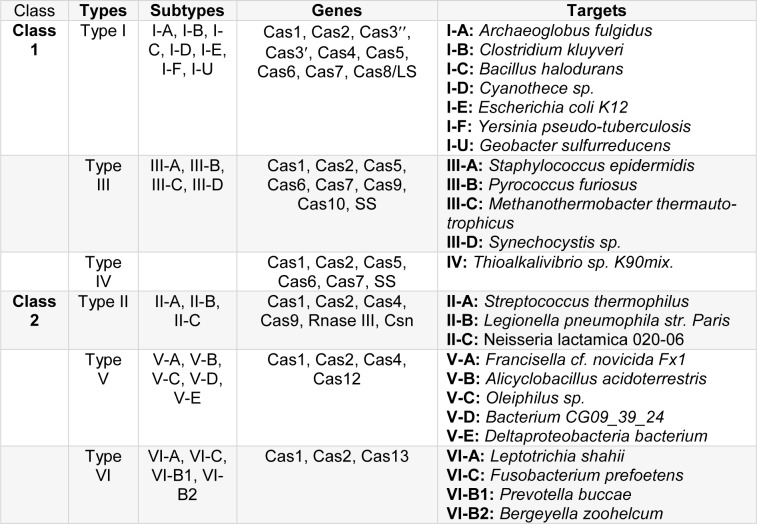
Organization of CRISPR-Cas types

**Table 2:**
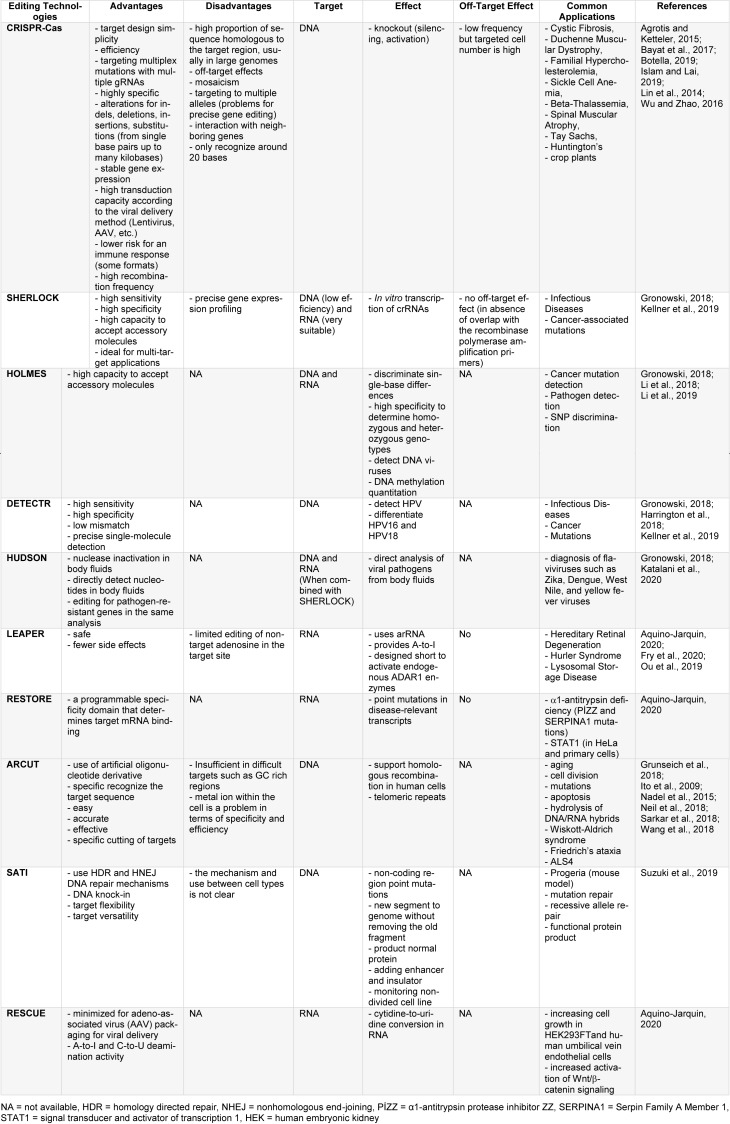
Advantages and disadvantages of genome editing techniques
